# Metrics and methods for characterizing dairy farm intensification using farm survey data

**DOI:** 10.1371/journal.pone.0195286

**Published:** 2018-05-09

**Authors:** Alejandra Gonzalez-Mejia, David Styles, Paul Wilson, James Gibbons

**Affiliations:** 1 SENRGy, Bangor University, Bangor, United Kingdom; 2 School of Biosciences, University of Nottingham, Sutton Bonington Campus, Sutton Bonington, United Kingdom; University of Illinois, UNITED STATES

## Abstract

Evaluation of agricultural intensification requires comprehensive analysis of trends in farm performance across physical and socio-economic aspects, which may diverge across farm types. Typical reporting of economic indicators at sectorial or the “average farm” level does not represent farm diversity and provides limited insight into the sustainability of specific intensification pathways. Using farm business data from a total of 7281 farm survey observations of English and Welsh dairy farms over a 14-year period we calculate a time series of 16 key performance indicators (KPIs) pertinent to farm structure, environmental and socio-economic aspects of sustainability. We then apply principle component analysis and model-based clustering analysis to identify statistically the number of distinct dairy farm typologies for each year of study, and link these clusters through time using multidimensional scaling. Between 2001 and 2014, dairy farms have largely consolidated and specialized into two distinct clusters: more extensive farms relying predominantly on grass, with lower milk yields but higher labour intensity, and more intensive farms producing more milk per cow with more concentrate and more maize, but lower labour intensity. There is some indication that these clusters are converging as the extensive cluster is intensifying slightly faster than the intensive cluster, in terms of milk yield per cow and use of concentrate feed. In 2014, annual milk yields were 6,835 and 7,500 l/cow for extensive and intensive farm types, respectively, whilst annual concentrate feed use was 1.3 and 1.5 tonnes per cow. For several KPIs such as milk yield the mean trend across all farms differed substantially from the extensive and intensive typologies mean. The indicators and analysis methodology developed allows identification of distinct farm types and industry trends using readily available survey data. The identified groups allow the accurate evaluation of the consequences of the reduction in dairy farm numbers and intensification at national and international scales.

## Introduction

Globally, dairy production emits 2,128 Mt CO_2_e yr^-1^ (roughly 5% of global anthropogenic emissions) and is responsible for a large share of environmental burdens including nutrient losses to air and water, water consumption and land use [1]. Demand for dairy products is rising which will lead to a further increase in burdens unless production efficiency increases. One route to this is to reduce land-use intensity of milk production by increasing milk yields per cow [2]. However, without advances in technology an environmental gain will only be achieved if the increase in production per cow out paces the increase in demand.

Despite already high milk yields per cow observed in many industrialised countries such as the United Kingdom (UK), dairy production continues on a long-term trend of reduction in farm numbers (consolidation) and intensification (C&I) that is driven by socio-economic and policy factors [3]. The UK dairy industry is the 10^th^ largest global producer of cow milk (accounting for 2.2% of world production) [4] and an exemplar of worldwide intensification trends. Between 2001 and 2014, the number of dairy farms in England and Wales decreased by 49%, from 20,191 to 10,274 [5], and the number of dairy cows decreased by 18%, whereas the average number of dairy cows per holding increased by 54%, from 87 to 134 [6], and the average annual milk yield (litres/cow) increased from 6,346 to 7,897 [7]. In other words, many farms have exited the sector, whilst remaining farms have grown in size and implemented more intensive practices that support higher milk yields. This trend is expected to continue following the abolition of milk quotas in 2015. However, there is little published information on changes in management and key performance indicators (KPIs) across individual farms, or types of farms, associated with this trend [8]. Alvarez et al. [9] emphasize the importance of finding the relationship between intensification and efficiency of dairy farming, and note the lack of studies researching dairy farm heterogeneity hidden behind sectoral statistics.

There is high variance in apparent dairy farm management efficiency, as indicated by KPIs such as nutrient use efficiency [10] and grass utilisation efficiency (the proportion of grass grown that is used by dairy cows [11]). Given this range in efficiency it might be expected that intensification of dairy farms will result in more efficient farms growing at the expense of less efficient farms. However, expanding French beef farms are becoming less economically efficient [12] because, despite an increase in investment in capital, technology and concentrate feed output has remained constant since 1990.

There are multiple measures of intensification such as the increase in farm output, herd size, feed concentrate use per unit of land or per head, produce per head and produce per unit of land [13]. Individually these indicators do not capture all dimensions of farm intensification and do not reflect the sustainability of that intensification [14]. Previous studies have assessed aspects of intensification and sustainability [15,16] through the application of productive efficiency methods such Stochastic Frontier Analysis [17,18] or the non-parametric method Data Envelopment Analysis [19]. There remains a need to characterise farm intensification beyond these economic and technical efficiency metrics in order to evaluate sustainable intensification.

One suggestion[20] is representing dairy systems with multiple derived variables that can be evaluated through the application of Principal Component Analysis (PCA) and clustering analysis. Clustering analysis has previously been applied to i) investigate whether intensification could improve the economic efficiency of dairy farms [9], ii) to classify dairy systems and compare them in terms of productivity, milk destination, maintenance of livestock biodiversity, land management, and landscape conservation [21], and iii) to explore social aspects such as factors that are relevant to quality of life for family dairy farms [22]. Here we build on these previous PCA and clustering approaches, using more robust statistical methods, to define dairy farm typologies according to wider socio-economic characteristics and physical parameters that can be linked to environmental performance and the derivation of carbon, land and nutrient footprints and potentially wider indirect (global) impacts.

We employ KPIs derived from detailed farm survey data to characterize dairy farm production and C&I. Consolidation is measured by the annual reduction in UK dairy farm numbers, and the sustainability of intensification is assessed in terms of physical and socio-economic characteristics critical to environmental, social and economic dimensions of sustainability, including: land use (e.g. grass and fodder) and tenure (i.e. owner occupied area), concentrate feed use, labour intensity, herd size and densities, productivity (i.e. milk yield), and milk price premium received.

### Methods

We used all available England & Wales Farm Business Survey (FBS) dairy farm data, providing 728 dairy farms in 2001 (out of a total all-farm survey population of 2845 across all farm types) declining to 432 farms in 2014 (out of a total all-farm survey population of 2447). These data are available under special license from the UK Data Archive [23–36]. Based on KPIs we identified major typologies of farms based on PCA and Clustering Analysis and then investigated how these KPIs and typologies have changed over a 14-year period characterized by structural change. We restricted our sample to farms that had on average at least 10 dairy cows in a calendar year. We then examined relationships among KPIs to identify groups of KPIs that measure particular aspects of farm structure. We also assessed whether relationships among KPIs remain constant over time i.e. if relationships were influenced by structural change (significant differences). The sample was then classified with a model-based clustering method that identified cohorts of similar dairy farms. We then examined changes in these cohorts (clusters) over the study period, 2001 to 2014.

### Farm survey data

Data representing physical-environmental and socio-economic characteristics of dairy farm businesses in England and Wales were extracted from the annual FBS, UK feed [37] and milk prices [38] from 2001 to 2014. Forty-eight variables were extracted annually to calculate 16 KPIs from 7281 farm business observations over 14 years of study. A total of 349,488 data points were analysed. The sample number of cows accounted for in the annual FBS data represents 4–5% of the dairy cow population in Wales and England (2001–2014). See S1 Table for summary of farms included.

The FBS was selected as a data source because it is a comprehensive source of information on socio-economic and physical characteristics of farms including labour, crops (previous and current harvest year, set-aside, by-products, forage and cultivations), livestock (cattle, dairy and other), costs (variable and fixed), assets, enterprise outputs, margins, and incomes. This authoritative source of information is based on a uniform sampling rate that ensures adequate coverage for analysis. Over the sample period farms remained in the survey for up to 15 years, with a replenishing rate of roughly 10% [39].

### Key performance indicators

We developed an approach to characterize dairy farms based on physical characteristics and production parameters that can be easily derived from farm survey data (Table 1). Our farm characterization is based on widely used variables and indicators that have been applied to represent the structure of dairy farming, its efficiency and the effects of C&I in the dairy business. We developed a set of KPIs using the underlying FBS survey data, but maximised information by transforming descriptors into quantities directly related to measures of production intensity, efficiency and other farm characteristics. We largely excluded economic parameters related to input and output prices, which are exogenous to the farms, but did include a measure of relative price received for milk (an indicator of a milk price premium). The KPIs were derived from widely used indicators to evaluate performance i.e. herd size, stocking rate, herd replacement rate, milk yield, feed amount or cost per animal, and labour requirements [40–42]. We added additional indicators such as areas of grass, fodder and cash crops to provide information on land use and feeding strategies that can be used to characterise farms. The agricultural area was also divided into two main areas; one utilised exclusively to grow and harvest crops for human consumption namely, “cash crop”, and the “non-cash crop” that is mainly for animal maintenance and that includes fallow, permanent and temporal grass (hay, silage, and grazing including rough grazing), silage cereals, and fodder crops (e.g. roots, kale, and maize) areas. The selected KPIs represent important characteristics of dairy farms with respect to sustainability and intensification, whilst avoiding duplication of information. To give equal weight during the statistical analysis, the KPIs were scaled by the annual mean value for each parameter but results are back scaled and presented in the original KPI units.

**Table 1:** Key performance indicators derived from FBS statistics in order to compare the intensity of production and characteristics among farms.

Farm metric		Units	Formula and description	Application
**Milk Production**	Total dairy cows	qty	Number of dairy cows	Herd size comparison
	Milk yield	l/ qty	Milk production / Dairy Cows	Measure of production efficiency. Higher yield generally means less inputs per production unit
	Milk premium	£/l ⁄ £/l	Milk Product Revenue / (Milk Products Sold *Average Milk Price)	Milk price received by farm compared to other farms. Premium >1 is desirable and <1 non-desirable
	Concentrate fed	tonne/ LU	Concentrate Feed Cost / (Concentrate Price * animals in Livestock Units (LU))	Feed bought into the farm that embodies upstream land and environmental impact (e.g. resource depletion, GHG emissions) per livestock unit
	Fodder fed	tonne/ LU	Coarse Fodder Cost / (Fodder Price * animals in Livestock Units (LU))	Measure of feed bought into the farm that embodies upstream land and environmental impacts (e.g. resource depletion, GHG emissions) per livestock unit
**Intensity of Livestock Production**	Cow fraction	qty/ LU	Dairy Cows / All animals in Livestock Units (LU)	Indicates the degree of the specialization and heterogeneity of the livestock enterprise.
	Cow stocking rate	LU/ ha	Cattle in Livestock Units (LU) / Non-Cash Crop Area	Measure of overall farm land use intensity. Useful for characterising farms and comparing management practices
	Livestock density	qty/ ha	Dairy Cows / Non-Cash Crop Area	Measure of land use intensity for dairy cows
	Labour intensity	hours/ ha	Annual worked hours / Farm Area	Indirect measure of technology. Useful for comparing farm productivity, and for socio-economic characterisation
**Grass, Fodder and Maize mix**	Fodder area	ha/ ha	Fodder Area /Grass Area	Measure of the reliance on fodder in feeding strategy. Could be used for inferring indoor/outdoor systems and land use footprints.
	Grass area	ha/ ha	Maize Area/Grass Area	Measure of maize dependence in feeding strategy. Could be used to infer land use footprints.
**Farm Structure for Animals**	Non-cash crop area in agricultural area	ha/ ha	Non-Cash Crop Area / Agricultural Area	Measure of farm livestock specialisation
	Grass area in agricultural area	ha/ ha	Grass Area / Agricultural Area	Measure of grass dependence in feeding strategy. Could be used for inferring indoor/outdoor systems. Useful for comparing farm land use footprints
**Production Area**	Production area	ha/ ha	Agricultural Area / Farm Area	Measures proportion of farm used for agricultural production.
**Tenure**	Tenure	ha/ ha	Owner Occupied Area / Agricultural Area	Measure of ownership structure and socio-economic characterisation.
**Replacement Rate**	Heifers	qty/ qty	Heifers / Dairy Cows	Measure of non-productive herd

### Data analysis

**Fig 1:**
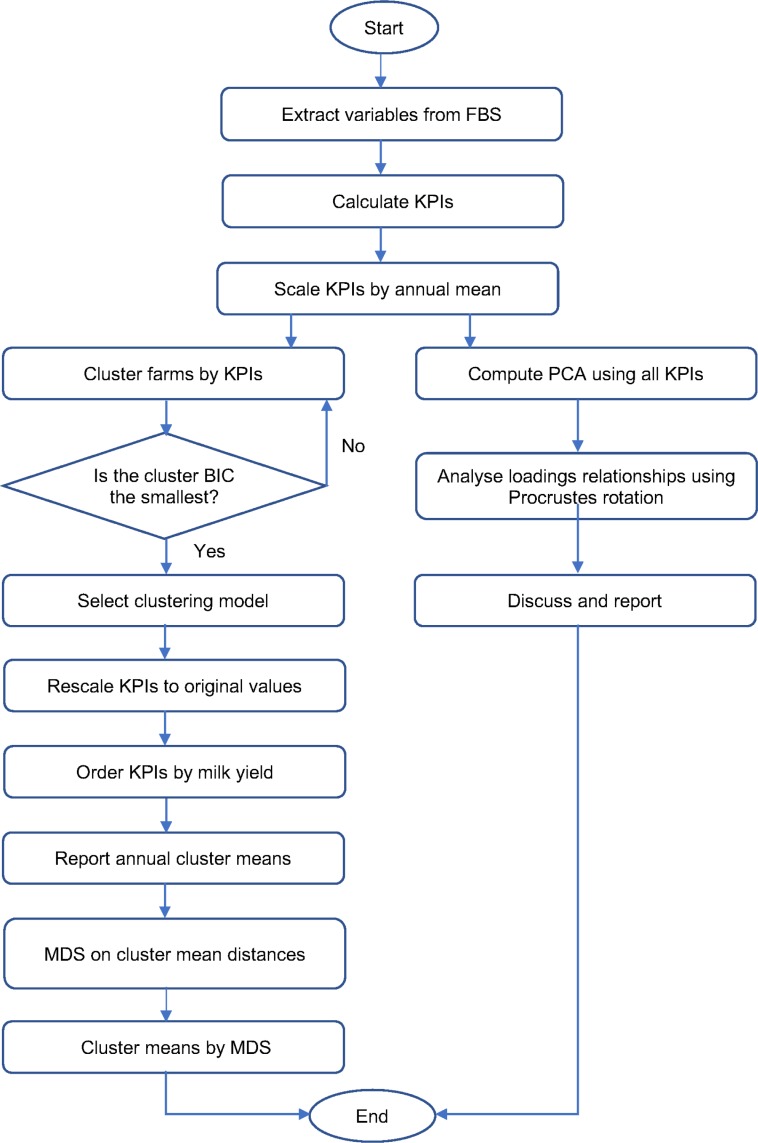
Statistical workflow used to analyse the key performance indicators (KPIs). - Number of clusters selected was determined by BIC (Bayesian Information Criterion).

We use a suite of statistical methods and workflow to analyse the data as shown in Fig 1. Further details of all the analysis methods with illustrations on simple data sets are available in [43,44] in particular we recommend chapter 14 of Hastie et al. All code to reproduce the data analysis is available on request from the authors. PCA (principal components analysis) was used to explore the relationship among KPIs (i.e. identification of fundamental farm properties) and how these relationships change over time. The usual aim is to reduce multiple dimensions down to two or three for illustration and analysis purposes. PCA creates new linear combinations of existing variables (components) ranked to explain as much variation as possible. The relative weighting of each KPI on each component is then termed the loading and value each farm on the component the score. For the set of KPIs to be a useful measure of farms over time, the relationship between KPIs should be relatively constant but change should result in farms changing their position along the KPI dimensions. PCA was calculated in R [45] and Procrustes rotation of the first 3 KPI loadings identified by PCA was used to compare the structure of each year and compare structure between years with the *vegan* package [46]. The Procrustes analysis rotated the PCA loadings to minimize the sum of squares of the difference in distance between loading for each year pair, a small total sum of squares indicating the relationship between the individual KPIs between years was similar, a large difference that the relationship changed between years. The rotation is necessary to fairly compare between years as the relationship between the variables and hence the relative loadings may remain constant over time but the absolute loadings may change and the sign of component loadings is arbitrary (can be positive or negative depending on the algorithm or data used).

Farms were clustered using Gaussian mixture model-based clustering with the *mclust* package in R [47,48]. In this method data are considered to originate from a distribution that is a combination of two or more components (i.e. clusters). Each component is modelled by a Gaussian distribution that is characterized by a mean vector, a covariance matrix, and an associated probability in the mixture. Each data point has 16 dimensions (KPI values) with a probability of belonging to each cluster. The model parameters are estimated using the Expectation Maximization algorithm initialized by hierarchical model-based clustering. Each cluster is centred at the mean with increased density for points near the mean [49].

We selected this method because the traditional clustering methods (k-means etc.) are heuristic and are not based on formal models with little statistical guidance on number of clusters. Further, the implicit assumptions that clusters are spherical and of equal size are very restrictive when, for example, we might expect there to be small cluster for rarer farm types and larger cluster for common farm types. Trials of k-means and k-medoids clustering on farm survey data performed poorly with a very unstable number of clusters identified. Another advantage of the model-based method is the flexibility of selection for the groups made by geometric features (shape, volume, orientation) of each cluster, which are determined by the covariance matrix. Different model options in *mclust* package are represented by identifiers e.g.: EVI, VEV and VVV. The first identifier denotes volume (equal or variable size), the second shape (spherical or not) and the third orientation (aligned or not). Accordingly, E stands for “equal”, V for “variable” and I for “coordinate axes”. For example, EVI denotes a model in which the volumes of all clusters are equal (E), the shapes of the clusters may vary (V), and the orientation is the identity (I) or coordinate axes. If all clusters were EEE the results would be similar to k-means clustering. Maximum likelihood is used to fit all these models, with different covariance matrix parameterizations, for a range of components. The best model was selected using the Bayesian Information Criterion or BIC; a small BIC score indicates strong evidence for the corresponding model [47]. BIC here trades off degree of model fit against model complexity. Model complexity increases with number of clusters and varying shape, orientation and volume of each cluster.

As the clustering was performed independently by year we then used multidimensional scaling (MDS) in order to group similar clusters based on their mean values for each KPI over time and track temporal changes of the same group. We tested the number of dimensions required to well-represent the clusters in ordination space. In this space, clusters more similar in their mean KPI values were closer in terms of ordination distance. For display we ranked within-year clusters by milk yield within year, which means that e.g. cluster 1 in 2001 does not necessarily correspond to cluster 1 in 2002.

## Results

### Relationships among KPIs

**Fig 2:**
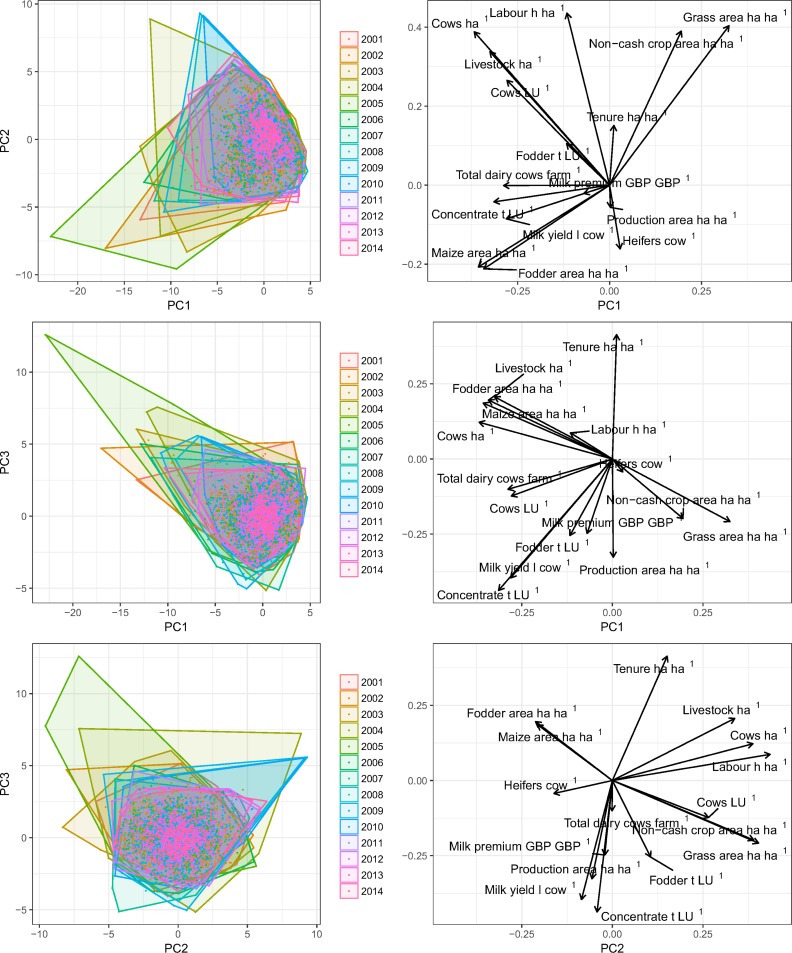
PCA results for all key performance indicator values across all years (2001–2014). Panels on the left show the PCA scores for individual farms, on the right loading for individual metrics.

The extracted time series from the FBS were used to compute KPIs that describe dairy farms in a 16-dimensional system (see S1 Fig for distribution). Annual PCAs were computed as well as a calculation that includes all data from 2001 to 2014 (Fig 2). Three dimensions of the PCA (PC1, PC2, and PC3) including all data sets from 2001 to 2014 explain approximately 50% of variation (S2 Fig). The loadings on the first 3 components broadly represent seven groups of KPIs (correlated in at least two components): i) milk production specifically (dairy cows, milk yield, concentrate feed per LU, and milk premium), ii) intensity and specialisation of livestock production (dairy stocking density, livestock density, dairy fraction, labour, and fodder per LU), iii) grazing prevalence (cash crop and grass presence), iv) grass/forage maize mix, v) production area, vi) tenure, and vii) replacement rate.

Area of land tenured by the owner of a farm is inversely related to dairy production area and replacement rate, which indicates that more heterogeneous farms with low replacement rates are more likely than more specialised dairy farms to be tenured by their owners (Fig 2).

The component scores in Fig 2 (left-hand plots) show that the majority of farms are concentrated at the centre of the axes for all years (2001–2014) with some outliers for years before 2006. There is some indication that there is less diversity in farms (points are closer together) in later years.

**Fig 3:**
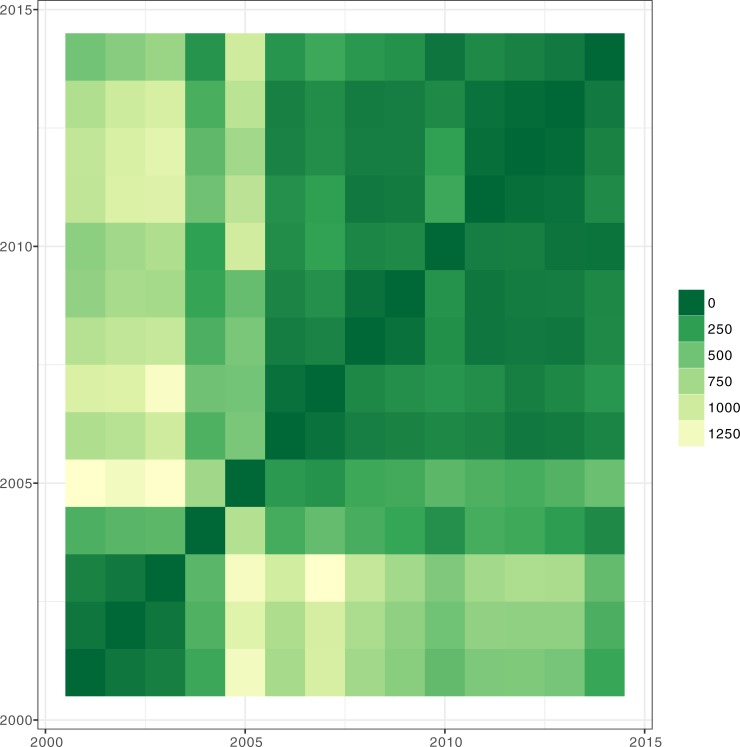
Procrustes analysis of annual variation in relationships among key performance indicators (KPIs) are derived from principle component analysis of annual data over the years 2001–2014, based on the sum of squared distances.

Procrustes rotation of the first 3 components (Fig 3) illustrates that in the periods 2001–2004 and 2006–2014 there are no large differences in the configuration of annual KPIs (sum of squares close to zero) while 2005 appears an outlier from all other years. This result suggests that the relationship between KPIs has largely remained stable over time, suggesting that they are reliable measures of farm properties even when structural changes are occurring.

### Cluster identification

Clustering analysis results indicate the number, configuration, and distinctiveness (mixing probabilities) of clusters for each of the survey years. Different cluster configurations are represented by the model i.e. VVV ellipsoidal, varying volume, shape, and orientation and VEV: ellipsoidal, equal shape. Number of farms decreased in the 14 years of study with the majority of farms distributed in mainly two or three clusters (higher probability). Further, clustering analysis identified three clusters for most years except for 2001 and 2003, which had four clusters, and 2011, 2012, and 2014, which had two clusters (Table 2). The distribution of farms among clusters was fairly even in most years with the exception of the smaller clusters (mixing probability < 0.1) (Table 2 & Fig 3). It is likely that these fluctuations in the smaller clusters are a combination of: (i) sampling artefacts where relatively rare farm configurations drop in and out of the sample; (ii) farms that are in transition, or: (iii) farms that have been affected by extreme events. In the majority of years, the individual clusters varied in volume, shape and orientation (VVV) although in a few years (2007, 2009, & 2010) clusters had equal shape (VEV) (Table 2).

**Table 2:** Clustering analysis results, indicating the number, configuration and distinctiveness (mixing probabilities) of clusters for each of the survey years.

Year	Cluster configuration	Number of clusters	log likelihood	n	df	Mixing probabilities			
						1	2	3	4
**2001**	VVV	4	1611	724	611	0.22	0.23	0.35	0.20
**2002**	VVV	3	431	678	458	0.50	0.48	0.02	
**2003**	VVV	4	862	643	611	0.38	0.30	0.30	0.02
**2004**	VVV	3	-182	512	428	0.48	0.37	0.16	
**2005**	VVV	3	32	477	458	0.42	0.52	0.06	
**2006**	VVV	3	393	464	458	0.42	0.35	0.23	
**2007**	VEV	3	67	469	428	0.46	0.42	0.12	
**2008**	VVV	3	337	493	458	0.55	0.42	0.03	
**2009**	VEV	3	366	488	428	0.47	0.44	0.09	
**2010**	VEV	3	623	479	428	0.40	0.15	0.45	
**2011**	VVV	2	390	479	305	0.37	0.63		
**2012**	VVV	2	454	467	305	0.44	0.56		
**2013**	VVV	3	1122	455	458	0.48	0.39	0.12	
**2014**	VVV	2	505	432	305	0.56	0.44		

**Fig 4:**
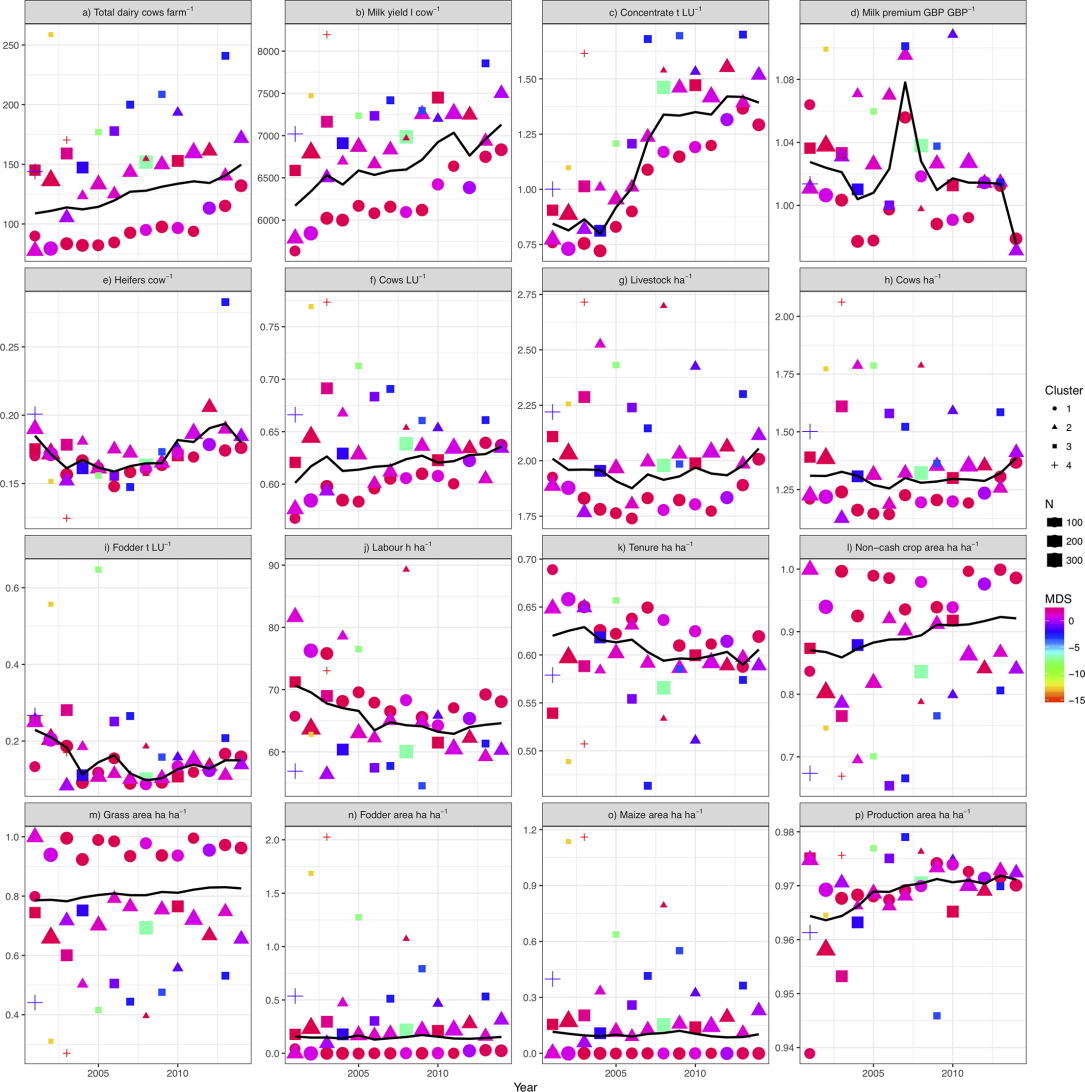
Trends in mean key performance indicator values for all identified clusters over the period 2001–2014. The number of farms in each cluster is represented by the size of symbol. Intensive systems are represented by triangles and extensive systems by circles. The solid black line represents the KPI annual average. The distance among all clusters in all years of study is represented by the colour scale MDS. This distance allows identifying which clusters are more similar.

Because the clustering analysis was performed for each year separately (a total of 41 clusters in 14 years of study, Table 2), we computed a MDS across clusters from all years in order to track the same type of cohort from one year to another. MDS in a single dimension had an almost perfect linear fit (R = 0.999) between the ordination distance and the observed dissimilarity. Thus, this second classification allows changes to be tracked over time for the same type of cluster, and the comparison of clusters. The results show two predominant clusters through time (groups 1 & 2) and other smaller groups (Fig 4). Since there are two main clusters from 2001 to 2014 and four more clusters that appear infrequently, we focused the following discussion on the two predominant clusters: group 1 (circles), classified as “*extensive systems”;* group 2 (triangles), categorized as “*intensive systems”* (Fig 4). Note that the outlying groups could comprise very intensive or “mega dairy” farms (Fig 4). All clusters are coloured by their MDS value; so similar clusters can be tracked over time and separated from less common typologies (Fig 4).

### Cluster comparison and trends

KPIs such as number of dairy cows, milk yield, concentrate use, grass in agricultural area, as well as productive area have increased in the 14 years of study (Fig 4); while the other KPIs have remained fairly constant, except for labour intensity, where lower labour use per hectare has been observed over time. Note that the black line that represents the KPI annual average at sectoral level rarely explains the actual value of a cluster for a particular year; extensive and intensive systems are generally above or below.

Fig 4B shows the difference in milk production between clusters. The intensive farms consistently produced more milk per cow than extensive systems. In 2014, there was an annual difference of 665 l/dairy cow in productivity and 8 hours/ha in labour intensity (Fig 4J). Intensive systems had an additional 40 dairy cows (Fig 3A) that consume 224 kg/yr more concentrate per LU (Fig 4C), 21 kg/yr less coarse fodder per LU (Fig 4I), and had a higher ratio of fodder and maize to grass (Fig 4N and 4O) (Table 3). There was no consistent difference in milk price premiums between intensive and extensive systems over the study period (Fig 4D), though intensive systems fared slightly better for more years.

**Table 3:** Comparison of key performance indicators (KPIs) between 2001 and 2014 for extensive (E) and intensive (I) farm cluster.

KPIs	E 2001	E 2014	I 2001	I 2014	E[2014–2001]	I[2014–2001]	2001 [E-I]	2014 [E-I]
**Total dairy cows**	78	132	145	172	55	27	68	40
**Milk yield**	5,784	6,835	6,588	7,499	1,051	911	-804	- 665
**Milk premium**	1.01	0.98	1.04	0.97	-0.03	-0.07	-0.03	-0.01
**Concentrate fed**	0.77	1.29	0.91	1.52	0.52	0.61	-0.13	- 0.22
**Fodder fed**	0.25	0.16	0.25	0.14	-0.09	-0.11	0.00	-0.02
**Cow fraction**	0.6	0.6	0.6	0.6	0.1	0.0	0.0	0.0
**Cow stocking rate**	1.9	2.0	2.1	2.1	0.1	0.0	-0.2	-0.1
**Livestock density**	1.2	1.4	1.4	1.4	0.1	0.0	-0.2	0.0
**Labour intensity**	82	68	71	60	-14	-11	10	8
**Fodder area**	0.0	0.0	0.2	0.3	0.0	0.1	-0.2	-0.3
**Grass area**	0.0	0.0	0.2	0.2	0.0	0.1	-0.2	-0.2
**Non-cash crop area in agricultural area**	1.0	1.0	0.9	0.8	0.0	0.0	0.1	0.1
**Grass area in agricultural area**	1.0	1.0	0.7	0.7	0.0	-0.1	0.3	0.3
**Production area**	0.97	0.97	0.98	0.97	0.00	0.00	0.00	0.00
**Tenure**	0.6	0.6	0.5	0.6	0.0	0.0	0.1	0.0
**Heifers**	0.2	0.2	0.2	0.2	0.0	0.0	0.0	0.0

Despite the difference in milk production, the replacement rate (Table 3), inferred from heifer to cow ratio (Fig 3E), has remained similar for intensive and extensive farms over the 14 years under study, initially declining for both clusters before increasing again towards a peak in 2012. Intensive farms have higher overall stocking densities (Fig 4G and 4H), but not necessarily a higher dairy fraction (3f) than extensive farms, though differences are small. In 2014, both systems had 1.4 dairy cows per hectare and 2 LU/ha (Table 3). Intensive and extensive systems can also be differentiated by the utilization rate of non-cash crop area (Fig 4L) and grass (Fig 4M) in the agricultural area of a farm, which have not changed dramatically since 2001 for each system (Table 3). Extensive systems utilised almost all non-cash crop agricultural area for grass production, compared to more intensive systems that only used 70% for grass production (Fig 4L and 4M and Table 3). Intensive farms produced maize on an area equivalent to 20% of grass area, and included fodder areas that grew over time, whilst extensive farms did not produce maize and are characterised by very small fodder areas compared to their grass extent (Fig 4O). There are a small number of intensive farms represented in the sporadic small clusters that appear in some years with comparatively very large maize areas (Fig 4O). Between 2001 and 2014, productivity (l cow^-1^) in extensive systems increased by 17% and cow numbers by 52%, compared with increases of 13% and 17% in productivity per cow and cow numbers, respectively, for intensive systems (Table 3). At the same time, labour intensity (hours/ha) has declined by 17–18% and the use of concentrate feed has increased by 500–600 kg/LU/yr in both intensive and extensive systems over the study period (Table 3). Across all farm types there was a large increase in the use of concentrates between 2005 and 2008 (Fig 3C).

Extensive systems were characterised by a 10% lower rate of owner occupation in 2001, which converged to a similar rate as for intensive systems in 2014, at around 60% of tenure (Fig 4K) (Table 3). Non-agricultural area, such as woodland, buildings, roads, water, and household gardens account for just 3% of farm areas across both intensive and extensive systems (Table 3).

## Discussion

### Identifying farm typologies and trends

Analysis of all dairy farm FBS data for the years 2001 through to 2014 confirms a trend of dairy consolidation [3], with a 41% decline in the number of dairy farms surveyed over that time period, and an 18% decline in dairy cow numbers—in line with separate statistics showing that the dairy cow population in England & Wales has declined by 19% over the same period. The total area represented by FBS dairy farms declined by just 7%, reflecting an increase in average dairy farm size from 132 ha in 2001 to 141 ha in 2014. Intensification is demonstrated by the 13–17% increase in milk production per cow over the same period. While the FBS data are drawn from a broadly representative set of farms recruited from agricultural census data there will inevitably sampling issues leading to the possible omission of rarer farm types such as very large and intensive dairy farms and farms following a more extensive pathway if these businesses choose not to be surveyed.

Many of the 16 KPIs evaluated changed significantly over the 14-year study period, reflecting productivity and efficiency improvements linked to diet change and technological advances. Consolidation is reflected in declining numbers of farms over a slightly declining aggregate area of dairy farms. Intensification is reflected in higher milk yields per cow, higher stocking rates and increased rates of fodder and concentrate feeding over time, coupled with a decline in labour intensity from 2001 to 2014 that presumably reflects technological improvements (e.g. investment in more efficient and automated milking parlours) and increased herd size linked to higher performing businesses remaining in dairy; this is in line with previous findings that have demonstrated the importance of efficient labour use in dairy [50,51].

We employed a methodology to characterise dairy farm typologies in England and Wales, underpinned by transformation of economic metrics reported in the FBS into KPIs that reflect physical and socio-economic characteristics of dairy farms. While similar approaches have been used previously our methodology is particularly robust because it combines multivariate analyses (PCA) with a Gaussian mixture model-based clustering and multidimensional scaling for grouping similar clusters over time. The method provides robust guidance on the number of clusters to choose. We found that the more usual clustering approaches (k-mean and k-medoid) did not perform well on these data which suggests their use may be inappropriate on FBS and similar data, especially when clusters are not expected to be of equal size or shape. This method can be applied to all agricultural sectors in all countries where farm economic statistics are compiled, providing a solid basis for rigorous and comprehensive evaluation of the sustainability of farm typologies and identified intensification pathways. This approach provides a robust basis for modelling the sustainability of pathways of intensification through time. For example, attributional LCA can be applied to determine the environmental footprints of milk production for statistically defined dairy farm typologies within (e.g. [52]) and between (e.g. [53]) years. Consequential LCA may also be applied to evaluate the environmental loading changes that arise, directly and indirectly, when farm typologies evolve in characteristics and predominance over time [54].

Clustering analysis of KPIs identified two main dairy farm typologies, representing different levels of intensification and following different but somewhat convergent pathways of intensification between 2001 and 2014. Results indicate that the dairy sector was more heterogeneous in the earlier years of the study, comprising three or four distinct clusters of dairy farms, and consolidated into just two main types of farm that predominated since 2011 (except for 2013). Classification of the two predominant farm typologies as “extensive” and “intensive” may appear to be a simplification, but these types are statistically identified and this does concur with a previous industry report [55] that identified two profitable pathways of dairy farm business development: (i) grass-based expansion to maximise margins per litre of milk; (ii) farm intensification to maximise margins per hectare. Intensive farms achieve higher milk yields per cow but use more concentrate feed and maize than extensive farms, which rely more heavily on grass and do not use maize. Smaller groups of farms identified in some years by clustering analysis had some characteristics indicative of very intensive farming methods, and may reflect a subset of emerging “mega-dairy” farms within the intensive cluster. Results also indicate some degree of convergence between the two main farm typologies, owing to a faster pace of intensification (e.g. increasing milk yield per cow) among the extensive farms. Consequently, in 2014, intensive and extensive farms had the same stocking densities per hectare.

### Evaluating the sustainability of intensification

Ongoing C&I is shifting UK dairy production away from small and medium sized farms towards larger farms that can be broadly categorised as grazing- or indoor- dominated systems. C&I pathways influence animal diets, health, yields, grassland and manure management, with implications for environmental and economic efficiency at animal, farm and system level. Whilst the definition of sustainable intensification is contested and may have different meaning in different contexts [56], a broad definition is to raise productivity and social welfare while reducing environmental impacts per unit of output. The measures captured through the use of farm survey data only include a small subset of those in a recent meta-analysis [14]. A more complete analysis would require socio-economic, biodiversity and soil health indicators. There is some evidence that environmental and economic indicators may be correlated but social indicators differ [50,57]. However, any regional or national analysis upscaling from farms requires being able to identify typologies of farms for which these indicators could be collected in a targeted manner. The indicators developed here can be linked to environmental performance, for example, feed strategies and land use that embody upstream land and environmental impact (e.g. land use, resource depletion, GHG emissions). Therefore, results of this research can be used to model scenarios including social aspects (e.g. labour intensity), economic components (e.g. profits per litres of milk), and environmental impacts (e.g. carbon, land and water footprints) of dairy farming. The clusters also provide a more accurate profile of trends in the sector than hitherto provided by analysis of “average” farms or aggregate data. There is also potential for application in terms of farm management as the developed KPIs could also be used to benchmark farms within cluster typologies, for example in terms of feed use efficiency, and to recommend priority practises to sustainably intensify that are targeted to the distinct cluster typologies.

We show that the UK dairy sector can be characterised by 2–3 clusters over the period of study, which allows environmental footprints to be readily calculated using LCA methods. Notably, across all clusters concentrate feed use, and by implication the indirect land footprint of dairy production, increased. A comprehensive analysis would require a wider system boundary than the individual farm. Increased maize and concentrate feed has the potential to improve animal-level efficiency and reduce on-farm environmental footprints [58,59], but may not reduce system level footprints owing to possible land use change GHG emissions [58–60]. Coupling the evolution of farm typologies described here with feed sourcing statistics and dairy-beef production models would enable a full LCA appraisal of direct and indirect environmental consequences arising from changes to animal diets and beef co-production [54]. Findings from this study may be directly transferable to dairy farming in other industrialised countries where similar C&I trends prevail.

Thus, this research provides a foundation for further analyses, in particular LCA [52,61] and DEA [62,63] that can address all aspects of sustainable intensification. Such work could represent a significant advance on previous studies of dairy intensification that have primarily focussed on environmental [52,53] or socio-economic factors [9,17–20] in isolation.

## Conclusion

Trends in dairy farm intensification are usually reported at the sectoral or “average farm” level, sometimes differentiated into regions or percentage quartiles, in terms of economic outputs, inputs, and margins [64]. Although useful for detecting broad trends, this approach does not adequately capture heterogeneity in farm operations, and does not relate business structure to the physical characteristics necessary to fully evaluate the sustainability of intensification trends. In the method developed here indicators calculated from farm business survey data coupled with robust model-based clustering identify the number of groups of farms and trends over time. We show that in England and Wales dairy farms have largely consolidated and specialized into two distinct clusters that now predominate within the sector: one “extensive” cluster of farms relying on expansion of grass-based milk production, with lower milk yields and labour intensity; one “intensive” cluster of farms producing, on average, more milk per cow with more concentrate and more maize, but fewer hours of labour per hectare. There is some indication that these clusters are converging as the extensive cluster is intensifying slightly faster than the intensive cluster, in terms of milk yield per cow and use of concentrate feed. The statistical characterisation of these groups will allow the accurate evaluation of the consequences of dairy C&I at national and international scales to be advanced.

## Supporting information

S1 Table Summary of FBS farms included in the analysis. (DOCX)

S1 Fig Summary boxplot of scaled metric data for each year analysed (2001–2014). (EPS)

S2 Fig Scree plot for PCA for all farms for all years showing a distinct “knee” after PC3. (EPS)
