# Supplementary material for: Metrics and methods for characterizing dairy farm intensification using farm survey data
Source: PLoS One. 2018 May 9;13(5):e0195286. doi: 10.1371/journal.pone.0195286 (PMC5942782; doi:10.1371/journal.pone.0195286)
Supplement: S1 Table — (DOCX) [file pone.0195286.s001.docx]

**Table S1. Summary of FBS farms included in the analysis**

| **Year** | **Number of farms all types** | **More than 10 dairy cows** | **% Dairy farms in FBS** | **FBS sample total dairy cows** |
| --- | --- | --- | --- | --- |
| 2001 | 2845 | 728 | 26% | 79,252 |
| 2002 | 2845 | 681 | 24% | 75,587 |
| 2003 | 2728 | 644 | 24% | 73,353 |
| 2004 | 2402 | 515 | 21% | 57,716 |
| 2005 | 2389 | 479 | 20% | 54,806 |
| 2006 | 2423 | 466 | 19% | 55,793 |
| 2007 | 2461 | 470 | 19% | 59,722 |
| 2008 | 2503 | 496 | 20% | 63,444 |
| 2009 | 2504 | 489 | 20% | 64,186 |
| 2010 | 2533 | 481 | 19% | 64,327 |
| 2011 | 2520 | 479 | 19% | 65,020 |
| 2012 | 2469 | 467 | 19% | 62,769 |
| 2013 | 2457 | 454 | 18% | 63,985 |
| 2014 | 2447 | 432 | 18% | 64,692 |
